# Outcomes in Pulmonary Arterial Hypertension: Results from a Single Center in Romania

**DOI:** 10.3390/jcm15207897

**Published:** 2026-10-13

**Authors:** Andrei Neagu, Ștefan Leu, Dragoș-Cosmin Zaharia, Claudia Lucia Toma, Roxana Enache, Daniel-Vlăduț Perpelea, Lucian Predescu, Pavel Platon, Miron Alexandru Bogdan, Tudor Constantinescu

**Affiliations:** 1 Department of Pulmonology, “Carol Davila” University of Medicine and Pharmacy, 020022 Bucharest, Romania; 2 Department of Pulmonology, “Marius Nasta” Institute of Pulmonology, 050159 Bucharest, Romania; 3 Department of Cardiology, “Carol Davila” University of Medicine and Pharmacy, 020022 Bucharest, Romania; 4 Department of Cardiology, “C. C. Iliescu” Emergency Institute for Cardiovascular Diseases, 022322 Bucharest, Romania; 5 Cardiac Catheterization Laboratory, “C. C. Iliescu” Emergency Institute for Cardiovascular Diseases, 022322 Bucharest, Romania

**Keywords:** pulmonary arterial hypertension, survival, renal function

## Abstract

**Background/Objectives**: Real-world data on pulmonary arterial hypertension (PAH) in Romania are scarce. This study evaluated the clinical characteristics, long-term survival patterns and the prognostic utility of baseline variables in a Romanian PAH cohort. **Methods**: We retrospectively analyzed adult patients treated for PAH at a single expert center between 2007 and 2026. The primary outcome was a composite of all-cause mortality or lung transplantation. Survival was assessed using Kaplan–Meier and univariable Cox proportional-hazard regression models. Univariable and multivariable linear regressions were used to identify drivers of baseline estimated glomerular filtration rate (eGFR). **Results**: The primary analysis cohort included 75 patients (mean age 53 ± 15 years, 71% female). Overall transplant-free survival was unexpectedly high at 97.2%, 80.8%, and 66.7% at 1, 3, and 5 years, respectively. The European Society of Cardiology (ESC)/European Respiratory Society (ERS) 3-strata risk score did not stratify survival (log-rank *p* = 0.9). Significant predictors of adverse outcomes in univariable analysis included six-minute walking distance (6MWD), PAH etiology and baseline coronary artery disease. In univariable analysis, lower baseline eGFR was associated with age, hypertension and diabetes mellitus, but not with pulmonary hemodynamics or transplant-free survival. **Conclusions**: In this regional PAH cohort, survival rates were high, likely reflecting survivor bias stemming from delayed access to care. Adverse outcomes were associated with PAH etiology and 6MWD, but the limited number of events precluded multivariable analysis.

## 1. Introduction

Pulmonary arterial hypertension (PAH) is a rare, progressive disease of the pulmonary vasculature characterized by the gradual onset of right heart failure [1,2]. Significant advances in PAH research over the past three decades have led to the development of a vast therapeutic arsenal [3]. This has allowed an improvement of median survival from 2.8 years in the pre-treatment era [4] to approximately 6 years in modern registries [5,6]. The choice of treatment strategy has a significant impact on survival in PAH [7]. For this reason, current management paradigms rely heavily on evidence-based therapeutic algorithms [8], where individualized risk stratification is essential for optimizing patient outcomes [9].

Prognostic assessment in PAH utilizes a suite of clinical and paraclinical variables associated with survival. While hemodynamic parameters obtained via right heart catheterization (RHC) remain the gold standard for baseline characterization, non-invasive markers—including World Health Organization functional class (WHO-FC), 6-min walking distance (6MWD), and circulating B-type natriuretic peptide (BNP) or its N-terminal fragment (NT-proBNP)—are increasingly relied upon for serial follow-up [1,2,9]. More recently, renal function, as measured by the estimated glomerular filtration rate (eGFR), has emerged as a promising non-invasive prognostic marker of clinical worsening and mortality in PAH and other forms of pulmonary hypertension (PH) [10,11,12,13,14].

Real-world PAH outcome data originate primarily from large national and international registries in North America and Western Europe [15,16], but there are significant regional disparities in terms of access to PAH medications and expert centers which may influence both diagnosis and prognosis [17]. In Eastern Europe, data on pulmonary vascular disease has largely come from retrospective or single-center studies [18,19,20] with only a few national registries in countries such as the Czech Republic [21] and Poland [22]. In this study, we report on the real-world experience of a dedicated PAH center in Romania, investigating baseline characteristics, survival patterns and the prognostic utility of both established risk stratification tools, as well as renal function.

## 2. Materials and Methods

### 2.1. Ethical Considerations

This study was conducted in accordance with the Declaration of Helsinki. All patient data were collected retrospectively and anonymized prior to analysis. The study was approved by the Ethics Committee of the “Marius Nasta” Institute of Pulmonology (approval number 12005/30 June 2026).

### 2.2. Study Population

We screened all patients who received treatment for PAH at the “Marius Nasta” Institute of Pulmonology between 2007 and April 2026. Patients were eligible for inclusion if they were ≥18 years of age at the time of diagnosis and had follow-up data available. PAH was defined per the 2022 European Society of Cardiology (ESC)/ European Respiratory Society (ERS) hemodynamic criteria (mean pulmonary artery pressure [mPAP] > 20 mmHg, pulmonary artery wedge pressure [PAWP] ≤ 15 mmHg and pulmonary vascular resistance [PVR] > 2 Wood units [WU]). Although this definition was not standard throughout the entire study period, we applied it uniformly in all cases. When PAWP exceeded 15 mmHg or was unavailable, left ventricular end-diastolic pressure (LVEDP) ≤ 15 mmHg was accepted as evidence of pre-capillary disease. Patients were excluded if they did not meet the hemodynamic definition or had missing data at baseline (defined as having <2 variables required for the ESC/ERS 3-strata risk score). The analysis was subsequently restricted to the subset of patients with serum creatinine measurements at baseline to evaluate the prevalence of renal dysfunction and to assess the prognostic value of eGFR.

All patients underwent high-resolution computed tomography and pulmonary function testing at diagnostic workup. Cases were adjudicated as Group 1 PAH by the treating clinician or multidisciplinary team when structural or functional pulmonary abnormalities were absent or mild relative to the severity of the hemodynamic findings. Patients in whom pulmonary or left heart disease was judged sufficient to explain the observed pulmonary hypertension were classified accordingly and excluded from this cohort.

This is a prevalent, retrospectively ascertained cohort rather than an inception cohort. We screened all patients ever treated for PAH at our center during the study period regardless of diagnosis date. Consequently, this methodology introduces a form of delayed entry that is not formally modeled in our survival analysis, since a distinct study-entry date independent of diagnosis date was not available.

### 2.3. Measurements

Demographic data, including age and sex, were abstracted from electronic medical records. Comorbidities were documented, including systemic hypertension, diabetes mellitus, coronary artery disease, atrial fibrillation, chronic obstructive pulmonary disease (COPD), and smoking status. Clinical variables recorded at baseline included WHO-FC and 6MWD. Biological variables included BNP or NT-proBNP, serum creatinine, and hemoglobin levels. Echocardiographic data included right atrial (RA) area, tricuspid annular plane systolic excursion (TAPSE), estimated systolic pulmonary arterial pressure (sPAP), left ventricular ejection fraction (LVEF), and the presence of pericardial effusion. Daily doses of loop diuretics and the use of mineralocorticoid receptor antagonists were recorded.

Hemodynamic parameters obtained by RHC included right atrial pressure (RAP), mPAP and PAWP. Cardiac output (CO) was measured using the indirect Fick method; cardiac index was calculated as CO divided by body surface area. The exact oxygen consumption estimation method could not be confirmed as uniform throughout the full study period. Pulmonary vascular resistance (PVR) was calculated as the pressure gradient (mPAP–PAWP) divided by CO. Stroke volume index (SVI) was calculated as cardiac index divided by heart rate. Mixed venous oxygen saturation (S_v_O_2_) was measured from pulmonary artery blood samples. Left ventricular end-diastolic pressure (LVEDP) was measured via left heart catheterization. Only baseline values were available for these parameters. Longitudinal follow-up measurements were not systematically recorded in this retrospective cohort.

Estimated glomerular filtration rate (eGFR) was calculated using the 2021 race-agnostic CKD-EPI equation [23]:

$$\mathrm{eGFR}=142\times {\min\left(\frac{S_{cr}}{\kappa },1\right)}^{\alpha }\times {\max\left(\frac{S_{cr}}{\kappa },1\right)}^{-1.200}\times 0.9938^{\mathrm{Age}}\times 1.012\;\left[\mathrm{if}\;\mathrm{female}\right]$$

where:

- eGFR is estimated glomerular filtration rate in mL/min/1.73 m^2^;
- *S_cr_* is standardized serum creatinine in mg/dL;
- κ = 0.7 (for females) or 0.9 (for males);
- α = −0.241 (for females) or −0.302 (for males).

Patients were categorized into 2 groups according to eGFR: ≥60 mL/min/1.73 m^2^ and <60 mL/min/1.73 m^2^. Where available, serum creatinine for baseline eGFR calculation was obtained during the diagnostic hospitalization for right heart catheterization, prior to initiation of PAH-specific treatment.

The ESC/ERS 3-strata risk score is calculated as the unweighted average of 18 potential component variables, each scored 1 (“low risk”), 2 (“intermediate risk”), or 3 (“high risk”) [1,2,9]. In this study, some of the 18 variables, such as those derived from cardiac magnetic resonance or cardiopulmonary exercise testing, were not systematically collected. The maximum number of variables available was 10: WHO-FC, 6MWD, NT-proBNP or BNP, RA area, the TAPSE/sPAP ratio, pericardial effusion, RAP, cardiac index, SVI and S_v_O_2_. As a data-completeness criterion, we set the minimum number of variables for each patient at 2; patients with <2 variables available were excluded. The unrounded average of available component scores was used to establish the final score: 1.0–1.49 (“low”), 1.5–2.49 (“intermediate”), and ≥2.5 (“high”).

### 2.4. Statistical Analysis

Continuous variables were expressed as mean ± standard deviation (SD) for normally distributed data, or median (interquartile range [IQR]) for skewed data. Categorical variables were expressed as absolute frequencies (*n*) and percentages (%). Comparisons between groups were performed using the Kruskal–Wallis rank-sum test for continuous variables and Pearson’s Chi-squared test for categorical variables. Fisher’s exact test was applied when categorical variable counts were low. Univariable and multivariable linear regression models were constructed to assess associations between eGFR as a continuous variable and other baseline parameters.

For survival analysis, the outcome was a composite of all-cause mortality or lung transplantation. Transplant-free survival time was calculated from the date of PAH diagnosis. Patients were censored at the last known visit, at 5 years of follow-up, or at the study cut-off date of 31 May 2026, whichever occurred first. Median follow-up was estimated using the reverse Kaplan–Meier (Schoenfeld) method. Survival was analyzed using the Kaplan–Meier method and the log-rank test. Univariable Cox proportional-hazard regression models were used to calculate hazard ratios (HRs). Proportional-hazard assumptions for univariable Cox models were assessed using Schoenfeld residuals. For variables with more than two levels (comorbidity burden, PAH classification, ESC/ERS 3-strata score, and WHO-FC), an overall *p*-value was calculated using the likelihood-ratio test; for continuous and binary variables, the *p*-value was derived from the Wald test, consistent with the reported hazard ratio and confidence interval. As a sensitivity analysis, survival and risk stratification analyses were repeated in the subset of patients meeting the hemodynamic definition of PAH via PAWP directly, excluding those confirmed only through the LVEDP fallback criterion.

All tests were two-sided, and a *p*-value < 0.05 was considered statistically significant. Analysis was performed in R version 4.5.1 (www.r-project.org, accessed on 1 July 2026).

## 3. Results

### 3.1. Baseline Characteristics

A total of 110 consecutive patients who received PAH treatment in our center were screened. After excluding patients who were <18 years of age at the time of PAH diagnosis or had no follow-up data available, hemodynamic confirmation of PAH was assessed in the remaining 100 patients; 76 met the hemodynamic definition. Of these, 75 had sufficient baseline data to calculate the ESC/ERS 3-strata risk score and constituted the primary analysis cohort. Among them, 54 had a serum creatinine measurement at the time of PAH diagnosis and constituted the secondary analysis cohort. Complete inclusion and exclusion criteria are presented in Figure 1.

The baseline demographic, clinical and paraclinical characteristics of the primary analysis cohort, stratified by the baseline ESC/ERS 3-strata risk score, are summarized in Table 1. The cohort was predominantly female (71%), with a mean age of 53 ± 15 years. Over half of the patients (52%) presented with at least one comorbidity at the time of PAH diagnosis, and 21% had a burden of two or more comorbidities. Idiopathic PAH (IPAH, 52%) and connective tissue disease-associated PAH (CTD-PAH, 33%) were the most frequent etiologies. Descriptive transplant-free survival differed substantially between these two predominant etiologies: patients with IPAH/heritable PAH (HPAH) had 1-, 3-, and 5-year survival of 97.3%, 94.1%, and 81.0%, respectively, compared with 95.8%, 63.7%, and 49.6% in patients with CTD-PAH. Most patients were clinically severe at the time of diagnosis, with 72% of them in functional class III/IV. The majority of patients were classified as intermediate risk on the ESC/ERS score (*n* = 56, 75%), while 12 patients (16%) and 7 patients (9%) met the criteria for low and high risk, respectively. The number of variables contributing to each patient’s ESC/ERS score ranged from 2 to 10 (median 7, IQR 5–9) across the primary analysis cohort, introducing potential heterogeneity in score precision, particularly for the two patients whose score was based on only 2 variables.

As expected, variables incorporated into the ESC/ERS risk calculation—including WHO-FC, 6MWD, NT-proBNP, RA area, TAPSE, sPAP, pericardial effusion, cardiac index and S_v_O_2_—differed significantly across the three risk strata. The prevalence of coronary artery disease was significantly increased in high-risk patients, as was the cumulative burden of comorbidities. While only 8.3% of low-risk patients had two or more comorbidities, this proportion rose to 71% in the high-risk stratum (*p* = 0.035). Baseline renal function did not demonstrate a statistically significant difference across the ESC/ERS risk groups. The prevalence of renal dysfunction, defined as eGFR < 60 mL/min/1.73 m^2^, was 14.8% (8 of 54 patients) in the renal analysis subset. Given this small numerator, analyses of dichotomized eGFR as a predictor (Table 2) are likely underpowered to detect a true prognostic effect and should be interpreted as exploratory.

Treatment intensity increased substantially over follow-up. At baseline, most patients were treated with monotherapy (65.3%), predominantly a phosphodiesterase-5 inhibitor or endothelin receptor antagonist, while 30.7% received double combination therapy and 2.7% received triple combination therapy. By the last available follow-up visit, monotherapy had decreased to 30.7% of patients, double combination therapy had increased to 40.0%, and 24.0% were receiving triple or quadruple combination therapy. Parenteral prostacyclin use (subcutaneous treprostinil or intravenous epoprostenol) increased from 2.7% at baseline to 6.7% at last follow-up. This escalation paralleled the expanding local availability of PAH-specific therapies during the study period: bosentan and sildenafil were available in Romania from 2008, macitentan and riociguat from 2016, and selexipag and subcutaneous treprostinil from 2022. Patients diagnosed in the earliest era (2007–2015, *n* = 17) were almost universally started on monotherapy (88.2%) but were frequently escalated to double or triple combination therapy by their last visit (64.7% combined), reflecting the introduction of newer therapeutic classes over the course of their disease. Patients diagnosed after 2022 (*n* = 34) had access to the full contemporary therapeutic armamentarium from baseline, though monotherapy remained the initial strategy in most cases (58.8%).

### 3.2. Survival and Prognostic Factors

Overall transplant-free survival for the primary analysis cohort was 97.2% (95% CI 93.4–100%) at 1 year, 80.8% (95% CI 71.5–91.3%) at 3 years and 66.7% (95% CI 54.8–81.2%) at 5 years. Median transplant-free survival was not reached within the 5-year observation window. During follow-up (median potential follow-up 5.0 years, 95% CI 3.8 years–not reached, estimated by the reverse Kaplan–Meier method), 18 of 75 patients (24.0%) reached the composite endpoint: 15 deaths and 3 lung transplantations. When stratified by baseline ESC/ERS score, survival did not differ significantly between risk strata and the pattern was not consistently ordered by risk category (Figure 2). Notably, the low-risk stratum did not show numerically higher 5-year survival than the intermediate-risk stratum (61.1% vs. 65.0%, Table S1), likely reflecting the small number of patients remaining at risk in each stratum by this time point rather than a true absence of a risk gradient. These estimates, and the wide confidence intervals surrounding them, should be interpreted with caution. As a sensitivity analysis, survival was reassessed in the subset of patients meeting the hemodynamic criteria for PAWP, without the LVEDP fallback (*n* = 71). Survival estimates in this restricted cohort were consistent with the primary analysis (97.0% [95% CI 93.0–100%] at 1 year, 81.1% [71.5–91.9%] at 3 years and 66.1% [53.7–81.2%] at 5 years) and the ESC/ERS 3-strata risk score similarly did not stratify transplant-free survival in this subset (log-rank *p* = 0.9).

To identify baseline predictors of transplant-free survival, univariable Cox proportional-hazard regression models were constructed using a priori selected clinical, hemodynamic, and echocardiographic variables (Table 2). In this analysis, coronary artery disease and a burden of ≥2 comorbidities emerged as significant predictors of adverse outcomes, although the overall comorbidity burden variable did not reach statistical significance (*p* = 0.100). Patients with associated PAH and PVOD had significantly lower transplant-free survival compared with IPAH/HPAH. Higher 6MWD was significantly protective, but hemodynamic variables did not reach statistical significance. Consistent with the Kaplan–Meier analysis, the baseline ESC/ERS 3-strata risk score did not reach statistical significance in the univariable Cox model (*p* = 0.850). Furthermore, baseline renal function, evaluated as a continuous eGFR variable, was not significantly associated with transplant-free survival. While these univariable associations provide valuable prognostic insight, the low overall number of clinical events in this cohort precluded the construction of multivariable Cox regression models, as adjusting for multiple confounders would violate the events-per-variable assumption and result in model overfitting. Proportional-hazard assumptions were satisfied for all univariable models (Schoenfeld residuals, global *p* > 0.05) with the exception of WHO-FC (global *p* = 0.007), indicating that its hazard ratio may not be constant over the follow-up period. This result should be interpreted with caution, though WHO-FC was not a statistically significant predictor in this analysis regardless (Table 2).

### 3.3. Baseline Renal Function: Predictors and Prognostic Value

When dichotomized at the clinically relevant threshold of 60 mL/min/1.73 m^2^, baseline eGFR did not significantly stratify transplant-free survival (Figure 3). To better understand the underlying drivers of renal function in this population, univariable and multivariable linear regression analyses were performed treating eGFR as a continuous outcome variable (Table 3 and Table 4).

In the univariable analysis (Table 3), advancing age, female sex, systemic hypertension and diabetes mellitus were strongly associated with lower baseline eGFR. 6MWD, NT-proBNP and right heart hemodynamic parameters, including RAP, cardiac index and PVR, did not demonstrate any statistically significant linear associations with eGFR.

Given the limited sample size of the renal analysis subset (*n* = 54), the multivariable model was restricted to the three univariable predictors with the strongest statistical associations—age, systemic hypertension, and diabetes mellitus (all *p* < 0.001)—to preserve an adequate observations-per-variable ratio and limit the risk of overfitting. Female sex, although also significantly associated with eGFR in univariable analysis (*p* = 0.024), was not included given its comparatively weaker association relative to the three selected predictors. After adjustment, diabetes mellitus remained a significant independent predictor of reduced eGFR. Advancing age retained a borderline association (*p* = 0.078), whereas systemic hypertension was no longer significant (*p* = 0.163).

## 4. Discussion

In this single-center retrospective study, we provide a real-world characterization of PAH in an Eastern European country which is under-represented in the literature. Three findings stand out. First, patients presented with high clinical severity at baseline, yet transplant-free survival was unexpectedly favorable. Second, the guideline-recommended ESC/ERS 3-strata risk score did not stratify transplant-free survival in this cohort. Third, outcomes were associated with both PAH-specific variables such as etiology, as well as systemic comorbidities. These findings carry implications for risk stratification in real-world, resource limited PAH populations. 

Comorbidities are frequent in real-world PAH cohorts, with 60–80% of patients presenting with at least one comorbidity at the baseline evaluation [24,25]. In our study, the prevalence of comorbidities was slightly lower at 52% (Table 1), likely due to the retrospective nature of the study. In terms of clinical severity, 72% of patients were in WHO-FC class III/IV at baseline and 74.7% were in the intermediate risk stratum of the ESC/ERS score, consistent with prior reports from large registries [26,27,28]. This high degree of clinical severity at baseline reflects the delays in diagnosis which characterize this rare disease [29,30,31]. The majority of patients were classified as IPAH and CTD-PAH. The proportion of patients with HPAH was very low, reflecting the limited availability of genetic testing for PAH. Patients classified as intermediate or high risk by the baseline ESC/ERS 3-strata score were characterized by a higher prevalence of systemic hypertension and coronary artery disease and a higher overall burden of comorbidities. Larger registries have also reported a higher prevalence of comorbidities in higher risk PAH patients [25,32].

Overall transplant-free survival was high in our cohort, reaching 97.2% at 1 year, 80.8% at 3 years, and 66.7% at 5 years. While modern therapeutic advances have improved the dismal pre-treatment era median survival of 2.8 years [4], the disease remains life-limiting, with larger registry studies reporting survival rates of 86–91% at 1 year, 67–78% at 3 years and 55–65% at 5 years [33,34,35,36,37]. Given the historical challenges and delayed access to PAH-specific therapies characteristic of our region, these favorable early outcomes are likely influenced by delayed entry inherent to our prevalent cohort design, a form of survivor bias whereby prevalent cohorts such as the one studied here tend to show better survival than incident cohorts [38]. In parallel, treatment intensity increased substantially over the study period: monotherapy at baseline fell to a minority of patients by last follow-up, with a corresponding rise in combination and parenteral prostacyclin therapy as newer therapeutic classes became available locally. This evolving treatment landscape, alongside survivor bias, likely also contributed to the favorable outcomes observed, particularly among patients diagnosed early in the study period who were escalated to combination therapy as it became available. We made efforts to identify and screen all patients who were diagnosed with and treated for PAH at our institution during the study period. However, due to limited access to expert PAH centers, patients with more severe forms of the disease may have died before a diagnosis could be established.

When evaluating the drivers of transplant-free survival in our cohort, PAH etiology and a cumulative burden of two or more comorbidities were among the variables associated with outcomes in univariable analysis, whereas individual hemodynamic and echocardiographic parameters did not reach statistical significance. Coronary artery disease was associated with a markedly higher risk of death or lung transplantation and patients with associated PAH or PVOD fared significantly worse than those with IPAH/HPAH, although the corresponding confidence intervals were wide (e.g., 95% CI 1.97–54.2 for PVOD), reflecting the limited number of events. A cumulative burden of two or more comorbidities showed a similarly elevated point estimate (HR 3.22, 95% CI 1.08–9.65), although the comorbidity burden variable did not reach significance as an overall predictor. A higher 6MWD was significantly associated with better survival, whereas echocardiographic and hemodynamic variables were non-significant. Additionally, the ESC/ERS 3-strata risk score did not stratify transplant-free survival in this cohort. Because the low number of events precluded multivariable adjustment, these univariable associations cannot account for potential confounding between predictors and should be interpreted as hypothesis-generating. They nonetheless highlight the potential role of comorbidities in the prognosis of PAH, a relationship that has been described in prior studies [25,39,40].

Right ventricular–pulmonary arterial (RV–PA) coupling has emerged as an important determinant of outcome in pulmonary hypertension, since RV function relative to afterload, rather than afterload alone, ultimately determines whether adaptive remodeling or maladaptive failure occurs. In a cohort of 65 patients with pre-capillary PAH, the ratio of RV free-wall longitudinal strain to systolic pulmonary artery pressure—a strain-based surrogate of RV-PA coupling—was an independent predictor of death or transplantation in multivariable analysis, outperforming conventional echocardiographic parameters [41]. This strain-based surrogate has since been validated against the invasive gold-standard measure of RV-PA coupling, the ratio of RV end-systolic to arterial elastance obtained by conductance catheterization [42]. TAPSE/sPAP, a more widely available surrogate that similarly integrates RV longitudinal systolic performance with pulmonary arterial afterload, has also demonstrated prognostic relevance in PAH, particularly for refining risk stratification among intermediate-risk patients [43]. In our cohort, TAPSE/sPAP was not significantly associated with transplant-free survival (HR 1.00 per 0.01 increase, 95% CI 0.95–1.05, *p* = 0.991). This null finding should be interpreted cautiously: TAPSE/sPAP was available in only 54 of 75 patients, and the small number of events in this subset limited statistical power considerably, consistent with the pattern seen across other echocardiographic and hemodynamic variables in our analysis. Given that the majority of our cohort was classified as intermediate risk by the ESC/ERS score—the group in which RV-PA coupling metrics appear most informative—the absence of a significant association in our analysis more plausibly reflects limited statistical power and incomplete echocardiographic ascertainment than a true absence of biological relevance. Strain-based coupling metrics such as RV free-wall longitudinal strain/sPAP were not available in our dataset. Larger, prospective studies incorporating these more granular measures alongside complete echocardiographic follow-up are needed to clarify the role of RV-PA coupling in real-world, resource-limited PAH populations such as ours.

One aim of this study was to evaluate the drivers and prognostic value of baseline renal function. Several prior reports have shown that lower eGFR at baseline is associated with poorer outcomes in PAH and other forms of PH [10,11,12,13,14]. While the mechanisms linking renal dysfunction and PH are complex and incompletely understood [44], the pulmonary hemodynamic modifications in PAH influence eGFR mainly through reduced kidney perfusion caused by lower cardiac output and through renal venous congestion caused by increased right heart filling pressures. These interactions have been described as the right-heart cardio-renal syndrome [45]. In our cohort, however, linear regression analyses revealed that baseline eGFR was significantly associated with age, systemic hypertension and diabetes in univariable analysis, but not with RAP and cardiac index. Diabetes remained a significant independent predictor in multivariable analysis. It should be noted that age is a direct term in the CKD-EPI equation used to calculate eGFR [23], so the association between advancing age and lower eGFR partly reflects this mathematical relationship rather than a physiological effect. Furthermore, because our multivariable model for eGFR included only the variables significant in univariable analysis (age, systemic hypertension, and diabetes mellitus), hemodynamic parameters were not tested within the same adjusted model. We therefore could not compare the prognostic relevance of comorbidities and hemodynamics for renal function. Baseline renal function—whether evaluated continuously or dichotomized at 60 mL/min/1.73 m^2^—was not significantly associated with transplant-free survival in our cohort (Figure 3 and Table 2). This null finding must be interpreted within the limitations of our sample size. The low overall number of clinical events severely constrained statistical power, a limitation also illustrated by the performance of the ESC/ERS 3-strata risk score (Figure 2). Taken together, our univariable findings are consistent with an association between eGFR and comorbidity burden in this cohort, but the study was insufficiently powered to definitively establish the independent prognostic utility of renal function or to support multivariable adjustments.

Several limitations must be acknowledged when interpreting the results of this study. First, as a retrospective observational study conducted at a single expert center, the analysis is inherently subject to missing data and unmeasured confounders. The exclusion of patients lacking sufficient baseline variables—while necessary for calculating the ESC/ERS risk score—introduces a degree of selection bias. Second, the hemodynamic definition of PAH was not uniform across the entire study period. We retrospectively applied the current hemodynamic definition to all patients, but some patients with borderline mPAP who were evaluated before 2022 would have been missed by screening. Third, CO was derived by the indirect Fick method—a recognized source of measurement error [46,47]. The exact protocol used to estimate oxygen consumption was not available for all RHC reports and may have varied across our extended study period, introducing additional imprecision into CO, cardiac index and PVR. Fourth, we cannot exclude the possibility that some patients may have been in a state of acute venous congestion at baseline, thus presenting transiently elevated creatinine values independent of the underlying chronic renal function. Fifth, longitudinal follow-up data were not systematically collected. We were therefore unable to assess how the treatment escalation observed over follow-up translated into functional or biochemical improvement, or to construct a serial, time-varying risk assessment. Finally, the small sample size combined with the low overall number of clinical events severely limited statistical power. This constraint prevented the construction of multivariable Cox proportional-hazard models in order to avoid overfitting and it likely contributed to a type II error regarding the prognostic performance of the ESC/ERS 3-strata score and baseline eGFR. Finally, because our cohort heavily reflects a delayed diagnosis phenotype characteristic of the local referral pathways, these findings may not be broadly generalized to PAH populations in regions with different demographic profiles or healthcare infrastructures with faster access to diagnostic and therapeutic resources for PAH.

## 5. Conclusions

In this single-center retrospective study from Romania, overall transplant-free survival for PAH patients was surprisingly high, a finding likely reflecting survivor bias inherent to a prevalent, retrospectively assembled cohort. The guideline-recommended ESC/ERS 3-strata risk score did not stratify transplant-free survival in this cohort. In univariable analysis, a comorbidity burden of two or more conditions, PAH etiology and 6MWD were associated with adverse outcomes, though these findings should be interpreted cautiously given the limited number of events, the wide confidence intervals around several estimates, and the absence of multivariable adjustment. Baseline renal function was associated with comorbidities but not with pulmonary hemodynamics and was not significantly associated with transplant-free survival in this cohort. These findings highlight the need for larger, prospective national registry studies in Romania to better understand real-world PAH outcomes and optimize local risk stratification.

## Figures and Tables

**Figure 1:**
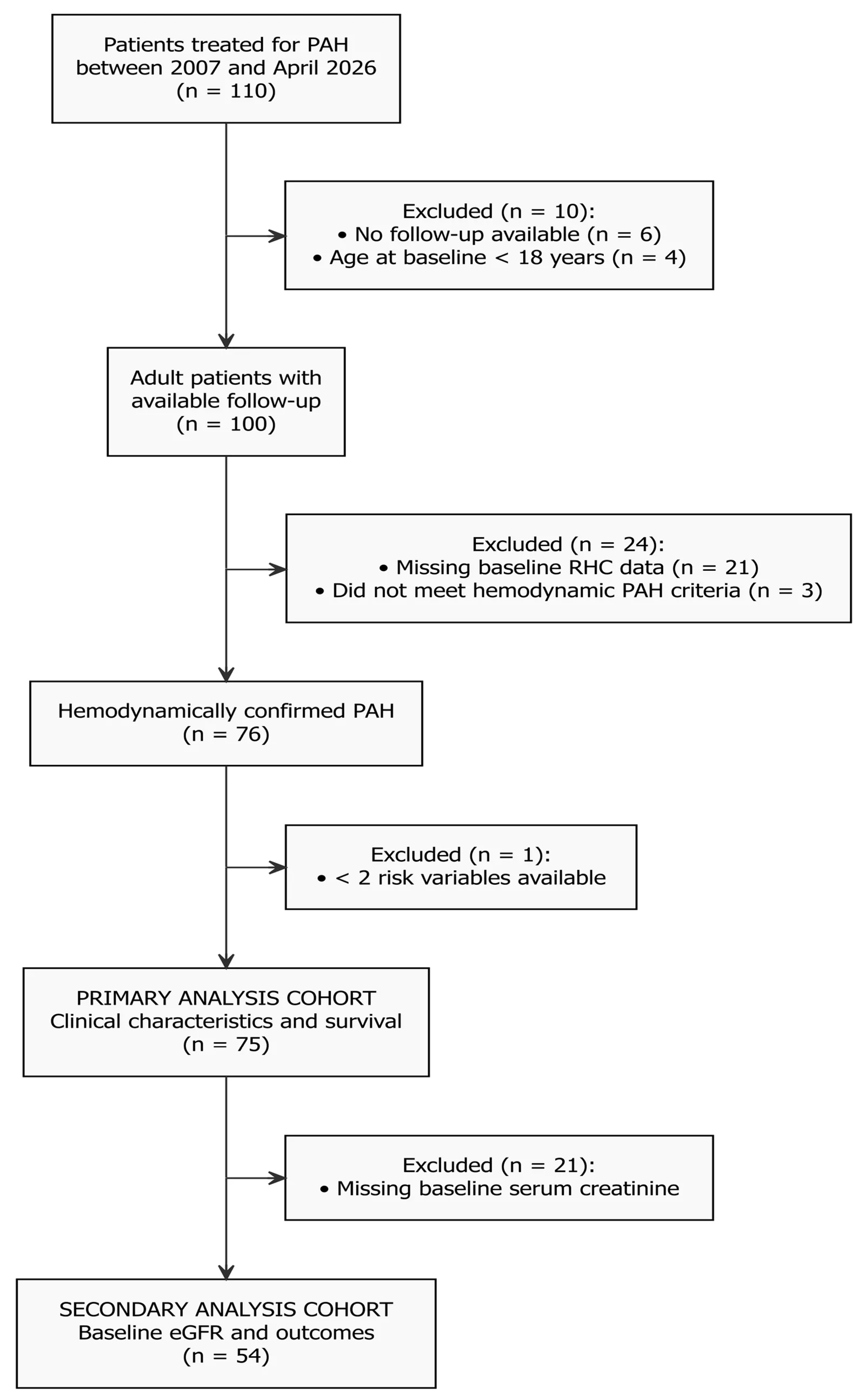
Flowchart detailing patient screening, inclusion, and exclusion criteria. Abbreviations: eGFR, estimated glomerular filtration rate; PAH, pulmonary arterial hypertension; RHC, right heart catheterization.

**Figure 2:**
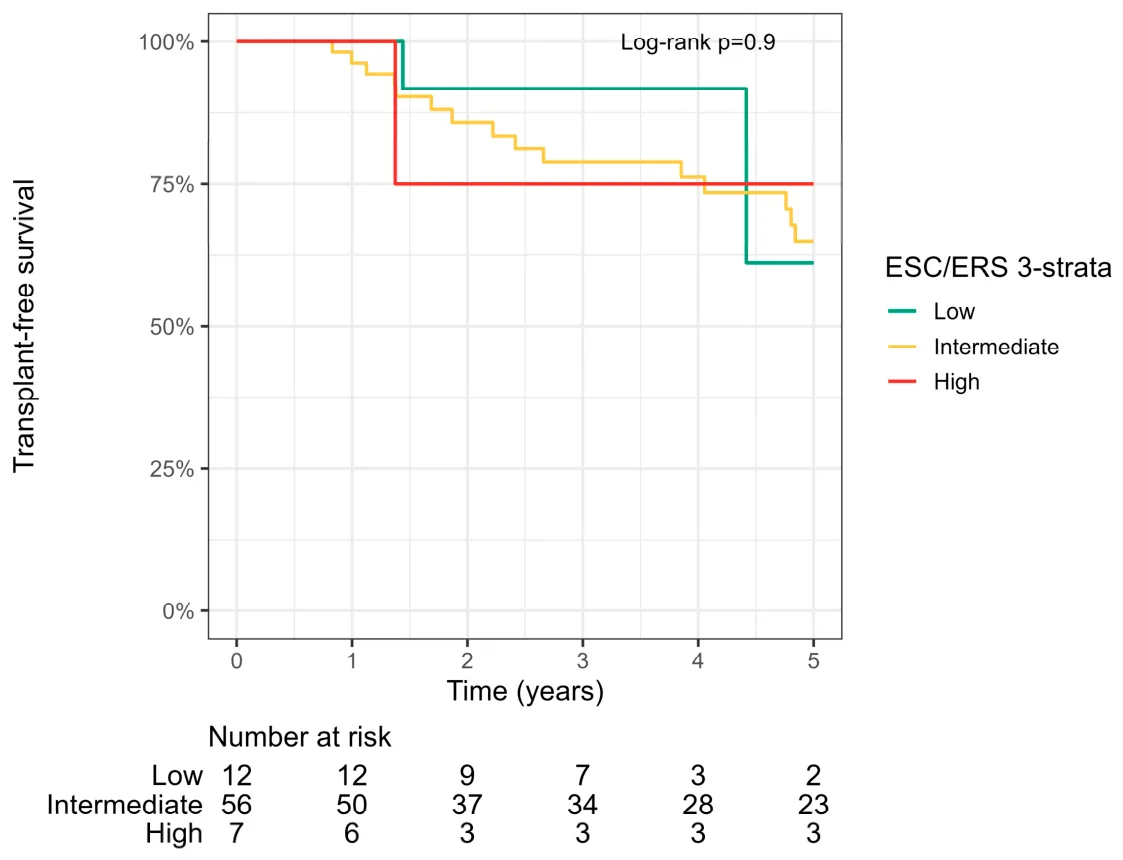
Kaplan–Meier curves showing transplant-free survival by ESC/ERS 3-strata risk at baseline. Abbreviations: ERS, European Respiratory Society; ESC, European Society of Cardiology.

**Figure 3:**
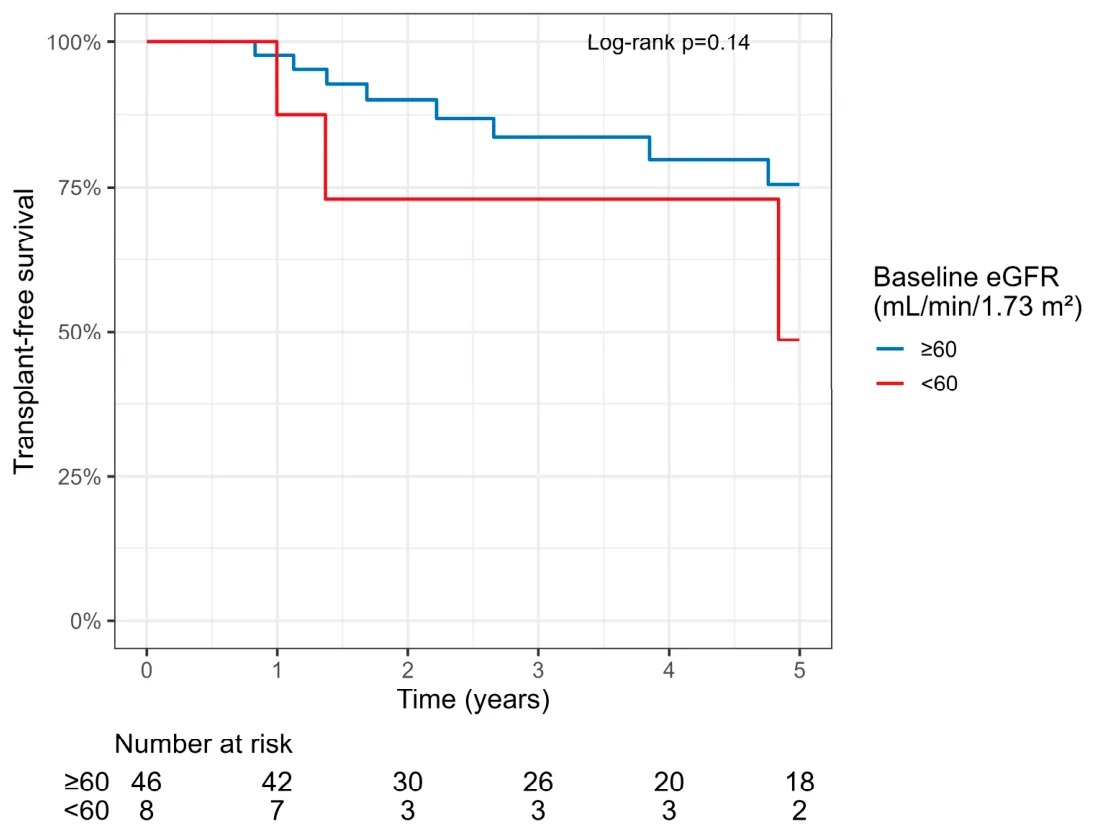
Kaplan–Meier curves showing transplant-free survival by baseline estimated glomerular filtration rate (eGFR).

**Table 1:** Baseline characteristics of the primary analysis cohort by ESC/ERS 3-strata risk.

Characteristic	*N*	Overall *N* = 75	Low *N* = 12	Intermediate *N* = 56	High *N* = 7	*p*-Value
Age (years)	75	53 (±15)	54 (±11)	52 (±16)	65 (±6)	0.14
Sex	75					0.5
Female		53 (71%)	7 (58%)	40 (71%)	6 (86%)	
Male		22 (29%)	5 (42%)	16 (29%)	1 (14%)	
Systemic hypertension	75	31 (41%)	6 (50%)	19 (34%)	6 (86%)	0.022
Diabetes mellitus	75	12 (16%)	0 (0%)	9 (16%)	3 (43%)	0.052
Coronary artery disease	75	9 (12%)	0 (0%)	6 (11%)	3 (43%)	0.027
Atrial fibrillation	75	4 (5.3%)	0 (0%)	3 (5.4%)	1 (14%)	0.4
COPD	74	6 (8.1%)	1 (8.3%)	4 (7.1%)	1 (17%)	0.6
Number of comorbidities	75					0.035
0		36 (48%)	6 (50%)	29 (52%)	1 (14%)	
1		23 (31%)	5 (42%)	17 (30%)	1 (14%)	
≥2		16 (21%)	1 (8.3%)	10 (18%)	5 (71%)	
PAH classification	75					0.7
IPAH		39 (52%)	4 (33%)	30 (54%)	5 (71%)	
HPAH		3 (4.0%)	1 (8.3%)	2 (3.6%)	0 (0%)	
PAH-CTD		25 (33%)	7 (58%)	16 (29%)	2 (29%)	
PAH-HIV		2 (2.7%)	0 (0%)	2 (3.6%)	0 (0%)	
PoPH		4 (5.3%)	0 (0%)	4 (7.1%)	0 (0%)	
PVOD		2 (2.7%)	0 (0%)	2 (3.6%)	0 (0%)	
WHO-FC	73					<0.001
I		1 (1.4%)	1 (8.3%)	0 (0%)	0 (0%)	
II		18 (25%)	7 (58%)	11 (20%)	0 (0%)	
III		51 (70%)	4 (33%)	43 (78%)	4 (67%)	
IV		3 (4.1%)	0 (0%)	1 (1.8%)	2 (33%)	
6MWD (m)	55	347 (±175)	512 (±118)	324 (±173)	269 (±103)	0.010
NT-proBNP (pg/mL)	37	1584 (±1923)	285 (±320)	1400 (±1460)	5055 (±2470)	<0.001
eGFR (mL/min/1.73 m^2^)	54	81 (±20)	93 (±18)	81 (±20)	65 (±19)	0.052
eGFR < 60 mL/min/1.73 m^2^	54	8 (15%)	0 (0%)	6 (14%)	2 (40%)	0.15
RA area (cm^2^)	36	21.8 (±5.0)	18.1 (±3.4)	21.8 (±4.6)	29.4 (±2.7)	0.012
TAPSE (mm)	60	17.7 (±3.7)	20.2 (±2.5)	17.4 (±3.7)	15.0 (±2.7)	0.010
Estimated sPAP (mmHg)	59	83 (±20)	53 (±13)	87 (±17)	97 (±15)	<0.001
Pericardial effusion	61					0.029
None		39 (64%)	9 (90%)	30 (63%)	0 (0%)	
Minimal		19 (31%)	1 (10%)	16 (33%)	2 (67%)	
Moderate or large		3 (4.9%)	0 (0%)	2 (4.2%)	1 (33%)	
RAP (mmHg)	59	6.5 (±3.8)	4.8 (±2.3)	6.6 (±3.7)	9.0 (±5.2)	0.2
mPAP (mmHg)	75	48 (±16)	37 (±17)	50 (±14)	59 (±18)	0.006
Cardiac index (L/min/m^2^)	66	2.21 (±0.64)	3.06 (±0.91)	2.08 (±0.46)	1.86 (±0.27)	<0.001
PVR (WU)	73	11.4 (±6.9)	5.5 (±4.0)	11.9 (±6.0)	17.2 (±10.4)	<0.001
S_v_O_2_ (%)	49	62 (±8)	73 (±2)	62 (±7)	53 (±6)	<0.001

Data are presented as n (%) or mean (±SD). *p*-values are calculated using the Kruskal–Wallis rank-sum test for continuous variables and Pearson’s Chi-squared test or Fisher’s exact test for categorical variables. Abbreviations: 6MWD, 6-min walking distance; COPD, chronic obstructive pulmonary disease; CTD, connective tissue disease; eGFR, estimated glomerular filtration rate; ERS, European Respiratory Society; ESC, European Society of Cardiology; HIV-PAH, pulmonary arterial hypertension associated with human immunodeficiency virus infection; HPAH, heritable pulmonary arterial hypertension; IPAH, idiopathic pulmonary arterial hypertension; mPAP, mean pulmonary arterial pressure; NT-proBNP, N-terminal pro-B-type natriuretic peptide; PAH, pulmonary arterial hypertension; PoPH, portopulmonary hypertension; PVOD, pulmonary veno-occlusive disease; PVR, pulmonary vascular resistance; RA, right atrium; RAP, right atrial pressure; sPAP, systolic pulmonary arterial pressure; S_v_O_2_, mixed venous oxygen saturation; TAPSE, tricuspid annular plane systolic excursion; WHO-FC, World Health Organization functional class; WU, Wood units.

**Table 2:** Univariable Cox proportional-hazard models predicting transplant-free survival.

Variable	*N*	HR	95% CI	*p*-Value
Age (per year)	75	1.03	1.00, 1.07	0.073
Sex	75			0.523
Female		—	—	
Male		1.36	0.53, 3.52	
Diabetes mellitus	75	2.13	0.60, 7.56	0.240
Coronary artery disease	75	4.46	1.26, 15.8	0.021
Atrial fibrillation	75	3.59	0.82, 15.8	0.091
Number of comorbidities	75			0.100
0		—	—	
1		1.00	0.32, 3.15	
≥2		3.22	1.08, 9.65	
PAH classification	75			0.011
IPAH/HPAH		—	—	
Associated PAH		3.44	1.19, 9.93	
PVOD		10.3	1.97, 54.2	
ESC/ERS 3-strata score	75			0.850
Low		—	—	
Intermediate		1.41	0.32, 6.25	
High		0.99	0.09, 11.1	
WHO-FC	73			0.398
I/II		—	—	
III		1.11	0.39, 3.17	
IV		6.45	0.68, 61.3	
6MWD (per 50 m)	55	0.85	0.73, 0.99	0.040
NT-proBNP (per 100 pg/mL)	37	1.01	0.96, 1.07	0.636
RA area (per cm^2^)	36	0.98	0.86, 1.12	0.799
TAPSE (per mm)	60	1.01	0.89, 1.15	0.878
TAPSE/sPAP (per 0.01)	54	1.00	0.95, 1.05	0.991
RAP (per mmHg)	59	0.97	0.84, 1.11	0.651
Cardiac index (per L/min/m^2^)	66	1.17	0.63, 2.19	0.620
PVR (per WU)	73	0.92	0.84, 1.01	0.067
eGFR (per mL/min/1.73 m^2^)	54	0.98	0.95, 1.01	0.254

Abbreviations: 6MWD, 6-min walking distance; CI, confidence interval; eGFR, estimated glomerular filtration rate; ESC, European Society of Cardiology; ERS, European Respiratory Society; HPAH, heritable pulmonary arterial hypertension; HR, hazard ratio; IPAH, idiopathic pulmonary arterial hypertension; NT-proBNP, N-terminal pro-B-type natriuretic peptide; PAH, pulmonary arterial hypertension; PVOD, pulmonary veno-occlusive disease; PVR, pulmonary vascular resistance; RA, right atrial; RAP, right atrial pressure; sPAP, systolic pulmonary arterial pressure; TAPSE, tricuspid annular plane systolic excursion; WHO-FC, World Health Organization functional class; WU, Wood units.

**Table 3:** Univariable linear regression models predicting baseline eGFR.

Variable	*N*	Beta	95% CI	*p*-Value
Age	54	−0.60	−0.92, −0.28	<0.001
Sex	54			0.024
Female		—	—	
Male		16	2.1, 29	
Systemic hypertension	54	−18	−28, −7.8	<0.001
Diabetes mellitus	54	−24	−37, −11	<0.001
6MWD (per 50 m)	42	1.8	−0.03, 3.7	0.053
NT-proBNP (per 100 pg/mL)	28	−0.21	−0.61, 0.19	0.297
RAP	42	0.63	−1.1, 2.4	0.472
Cardiac index	46	3.0	−6.4, 12	0.525
PVR	52	−0.24	−1.0, 0.52	0.529

Abbreviations: 6MWD, 6-min walking distance; CI, confidence interval; eGFR, estimated glomerular filtration rate; NT-proBNP, N-terminal pro-B-type natriuretic peptide; PVR, pulmonary vascular resistance; RAP, right atrial pressure.

**Table 4:** Multivariable linear regression model predicting baseline eGFR.

Variable	Beta	95% CI	*p*-Value
Age	−0.33	−0.70, 0.04	0.078
Systemic hypertension	−8.1	−19, 3.4	0.163
Diabetes mellitus	−16	−30, −3.4	0.015

Abbreviations: CI, confidence interval; eGFR, estimated glomerular filtration rate.

## Data Availability

The raw data supporting the conclusions of this article may be made available by the authors on reasonable request.

## Supplementary Materials

The following supporting information can be downloaded at: https://www.mdpi.com/article/10.3390/jcm15207897/s1, Table S1: Transplant-free survival estimates at 1, 3, and 5 years stratified by baseline ESC/ERS 3-strata risk.

## Abbreviations

The following abbreviations are used in this manuscript:

6MWD	6-min walking distance
BNP	B-type natriuretic peptide
CO	Cardiac output
COPD	Chronic obstructive pulmonary disease
CTD-PAH	Pulmonary arterial hypertension associated with connective tissue disease
eGFR	Estimated glomerular filtration rate
ERS	European Respiratory Society
ESC	European Society of Cardiology
HIV-PAH	Pulmonary arterial hypertension associated with human immunodeficiency virus infection
HPAH	Hereditary pulmonary arterial hypertension
IPAH	Idiopathic pulmonary arterial hypertension
LVEDP	Left ventricular end-diastolic pressure
LVEF	Left ventricular ejection fraction
mPAP	Mean pulmonary arterial pressure
NT-proBNP	N-terminal fragment of the prohormone of B-type natriuretic peptide
PAH	Pulmonary arterial hypertension
PAWP	Pulmonary artery wedge pressure
PoPH	Porto-pulmonary hypertension
PVOD	Pulmonary veno-occlusive disease
PVR	Pulmonary vascular resistance
RA	Right atrium
RAP	Right atrial pressure
RHC	Right heart catheterization
sPAP	Systolic pulmonary artery pressure
SVI	Stroke volume index
S_v_O_2_	Mixed venous oxygen saturation
TAPSE	Tricuspid annular plane systolic excursion
WHO-FC	World Health Organization functional class
WU	Wood units
