# Supplementary material for: Outcomes in Pulmonary Arterial Hypertension: Results from a Single Center in Romania
Source: J Clin Med. 2026 Oct 13;15(20):7897. doi: 10.3390/jcm15207897 (PMC13705510; doi:10.3390/jcm15207897)
Supplement: Supplementary file 1 [file jcm-15-07897-s001.zip › jcm-4524844-supplementary.pdf]

Supplementary Materials

Outcomes in Pulmonary Arterial Hypertension: Results from a Single Center in Romania

Andrei Neagu [1,2,*], Ștefan Leu [2], Dragoș-Cosmin Zaharia [1,2], Claudia Lucia Toma [1,2], Roxana Enache [3,4], Daniel-Vlăduț Perpelea [3,4], Lucian Predescu [3,5], Pavel Platon [5], Miron Alexandru Bogdan [1] and Tudor Constantinescu [1,2]

1 Department of Pulmonology, "Carol Davila" University of Medicine and Pharmacy, 020022 Bucharest, Romania
2 Department of Pulmonology, "Marius Nasta" Institute of Pulmonology, 050159 Bucharest, Romania
3 Department of Cardiology, "Carol Davila" University of Medicine and Pharmacy, 020022 Bucharest, Romania
4 Department of Cardiology, "C. C. Iliescu" Emergency Institute for Cardiovascular Diseases, 022322 Bucharest, Romania
5 Cardiac Catheterization Laboratory, "C. C. Iliescu" Emergency Institute for Cardiovascular Diseases, 022322 Bucharest, Romania
* Correspondence: andrei.neagu@drd.umfcd.ro

Table S1. Transplant-free survival estimates at 1, 3, and 5 years stratified by baseline ESC/ERS 3-strata risk.

Characteristic	1-Year Survival	3-Year Survival	5-Year Survival	p-value
ESC/ERS 3-strata				0.9
Low	100.0% (100.0%, 100.0%)	91.7% (77.3%, 100.0%)	61.1% (27.0%, 100.0%)	
Intermediate	96.2% (91.1%, 100.0%)	78.8% (67.9%, 91.5%)	65.0% (51.9%, 81.5%)	
High	100.0% (100.0%, 100.0%)	75.0% (42.6%, 100.0%)	75.0% (42.6%, 100.0%)	

Data are presented as percentage (95% confidence interval). The p-value was calculated using the log-rank test.
Abbreviations: CI, confidence interval; ERS, European Respiratory Society; ESC, European Society of Cardiology.
